# Genetic Variability, Population Differentiation, and Correlations for Thermal Tolerance Indices in the Minute Wasp, *Trichogramma cacoeciae*

**DOI:** 10.3390/insects12111013

**Published:** 2021-11-10

**Authors:** Michela Ion Scotta, Lucas Margris, Nadine Sellier, Sylvie Warot, Flavio Gatti, Fabio Siccardi, Patricia Gibert, Elodie Vercken, Nicolas Ris

**Affiliations:** 1 INRAE, Institut Sophia-Agrobiotech, Université Côte d’Azur, CNRS, 06903 Sophia Antipolis, France; lucas.margris@inrae.fr (L.M.); nadine.sellier@inrae.fr (N.S.); Sylvie.Warot@inrae.fr (S.W.); Elodie.Vercken@inrae.fr (E.V.); nicolas.ris@inrae.fr (N.R.); 2 Department of Physics (DIFI), University of Genoa, INFN, 16146 Genoa, Italy; flavio.gatti@ge.infn.it (F.G.); fabio.siccardi@ge.infn.it (F.S.); 3 CNRS, Laboratoire de Biométrie et Biologie Evolutive UMR 5558, University of Lyon, Université Lyon 1, 69100 Villeurbanne, France; patricia.gibert@univ-lyon1.fr

**Keywords:** chill coma temperature, activity recovery, critical thermal minimum, local adaptation, parasitoids, thermal tolerance indices, *Trichogramma*

## Abstract

**Simple Summary:**

Augmentative biological control relies on the more or less frequent/abundant releases of biological control agents (BCAs) that have to be adapted to their short-term local environment including (micro-)climatic conditions. Thermal biology of BCAs is thus a key component for their success. The extent to which thermal tolerance indices may be relevant predictors of the field efficiency is however still poorly documented. Within this frame, we investigated the intraspecific variability for the ability to move at low temperatures in the minute wasp, *Trichogramma cacoeciae*. We collected, molecularly characterized, and compared for their thermal tolerance indices numerous strains originating from three contrasting geographic areas. Our findings evidenced both a geographic differentiation between strains for one of the thermal tolerance indices and a positive correlation between two of them, demonstrating the existence of an intraspecific variability.

**Abstract:**

Temperature is a main driver of the ecology and evolution of ectotherms. In particular, the ability to move at sub-lethal low temperatures can be described through three thermal tolerance indices—critical thermal minimum (CTmin), chill coma temperature (CCT), and activity recovery (AR). Although these indices have proven relevant for inter-specific comparisons, little is known about their intraspecific variability as well as possible genetic correlations between them. We thus investigated these two topics (intraspecific variability and genetic correlations between thermal tolerance indices) using the minute wasp, *Trichogramma cacoeciae*. Strains from *T. cacoeciae* were sampled across three geographic regions in France—two bioclimatic zones along a sharp altitudinal cline in a Mediterranean context (meso-Mediterranean at low elevations and supra-Mediterranean at higher elevations) and a more northwestern area characterized by continental or mountainous climates. Our results evidenced a significant effect of both the longitude and the severity of the cold during winter months on CCT. Results were however counter-intuitive since the strains from the two bioclimatic zones characterized by more severe winters (northwestern area and supra-Mediterranean) exhibited opposite patterns. In addition, a strong positive correlation was observed between CCT and CTmin. Neither strain differentiation nor the covariations between traits seem to be linked with the molecular diversity observed on the part of the mitochondrial marker COI.

## 1. Introduction

The process of local adaptation is a central theme in evolutionary ecology and, among selective abiotic factors, temperature is thought to be of major importance, in particular for ectotherms [1,2]. Within this frame, environmental gradients represent ideal spatial organizations to investigate population differentiation related to thermal adaptation [3,4]. In particular, both latitudinal and altitudinal gradients usually offer a progressive variation of the mean ambient temperature along variable geographic distances and, thus, experimentally tractable case-studies [5,6]. Yet, some drawbacks—e.g., co-variation between temperature and other abiotic factors, and distinction between direct and indirect causes—have to be considered cautiously.

Low temperatures are a significant source of stress for many insect species and are supposed to limit fitness and ultimately the distribution of species [7,8]. One particular aspect of thermal adaption is the ability of individuals to maintain a locomotor activity at low temperatures, which can be described using three thermal indices: (i) the critical thermal minimum (CTmin), i.e., the temperature at which the individuals lose locomotor activity, (ii) the chill coma temperature (CCT), i.e., the temperature at which the individuals lose all movement, and (iii) the activity recovery (AR), i.e., the temperature at which animals recover locomotor activity. Several authors emphasize the relevance of these thermal tolerance indices to experimentally investigate inter- and, to a lesser extent, intra-specific variabilities [9,10,11,12,13,14,15]. At the intra-specific level, the extent to which these indices actually reflect local adaptations and how they covary remain however poorly documented apart from some “model organisms” such as *Drosophila* species. 

Besides academic purposes, eco-evolutionary processes affecting thermal biology are also of prime interest for more applied purposes, in particular in the frame of biological control—the use of living organisms (called biological control agents or BCAs) against noxious ones [16]. Incidentally, it is noteworthy that the “ins and outs” differ according to the biological control strategy. Because classical biological control aims at the long-term establishment of an exotic BCA, the adequacy of this latter towards the thermal environment in the future area of introduction is considered as a key parameter of its establishment success. Climate matching approaches are in such a case actually relevant (see, for instance, [17,18,19]) and some intra-specific genetic variations may indeed explain contrasted results [20]. However, because augmentative biological control (in particular the inundative sub-strategy) only aims at a transitory BCA presence and pest control, the thermal adequacy of BCAs has to be reflected and then optimized with regard to some particular (micro)climatic conditions. More precisely, those conditions may (i) only represent part of the seasonal variations (open field crops), (ii) be disconnected from the actual climate and artificially controlled (greenhouses productions), (iii) be modulated by the architecture and physiology of the plants (see, for instance, [21,22,23]), and/or (iv) be far from the BCA’s “comfort zone”. With regard to this last point, a relevant challenge is thus to choose or even genetically improve biological control agents that could be used at low temperatures early in the season in order to prevent pest outbreaks. To our knowledge, such attempts remain rare [24,25,26]. For macro-organisms used as biocontrol agents (e.g., predatory arthropods or insect parasitoids), one of the limits to this challenge is probably the absence of relevant and experimentally tractable proxies for evaluating the overall performances at low temperatures. We hypothesized that the thermal tolerance indices (critical thermal minimum, chill coma temperature, and activity recovery) may play the role of such proxies.

Given this context (both fundamental and applied research), we investigated the intra-specific differentiation for thermal tolerance indices as well as the correlation between these traits in the minute parasitic wasp, *Trichogramma cacoeciae* Marchal (Hymenoptera, Trichogrammatidae). As with other *Trichogramma* species, *T. cacoeciae* is an oophagous parasitoid whose biology and ecology can be summarized as follows [27]: The female wasp lays each of its eggs inside a host egg whose embryo is rapidly killed. The offspring’s pre-imaginal development then occurs within the host egg until metamorphosis and the emergence as an adult. In *T. cacoeciae*, reproduction is ensured through thelytoky (diploid females producing diploid daughters without fecundation by males); this parthenogenesis not being induced by heritable symbionts [28]. Because asexuality is traditionally considered as a desirable feature for biological control [29], *T. cacoeciae* has often been evaluated or even used as a biocontrol agent (see, for instance, [30,31,32]). Moreover, *T. cacoeciae* is known to be a widespread species and has been sampled repeatedly in Europe [33]. At a very local scale (south-eastern France), *T. cacoeciae* is also the most abundant species along an altitudinal gradient [34]. However, the relative contributions of recurrent recolonizations through active and/or passive dispersal and local adaptations to contrasted climates remain poorly investigated. It is worth noting that, at this scale, no use of *T. cacoeciae* for augmentation biological control is documented so that natural eco-evolutionary processes are not disturbed by external inputs of individuals. To test for the presence of local adaptation, we constituted a pool of *T. cacoeciae* strains collected at both macro- (latitudinal cline) and micro- (altitudinal gradient) scales. The strains were characterized genetically using part of the cytochrome oxidase I [35], and compared phenotypically using the three thermal tolerance indices: CTmin, CCT, and AR. We also investigated correlations between these three thermal tolerance indices in order to point out possible negative and positive correlations between traits that are, respectively, indicative of evolutionary constraints or strategies.

## 2. Materials and Methods

### 2.1. Terminology

**Figure 1:**
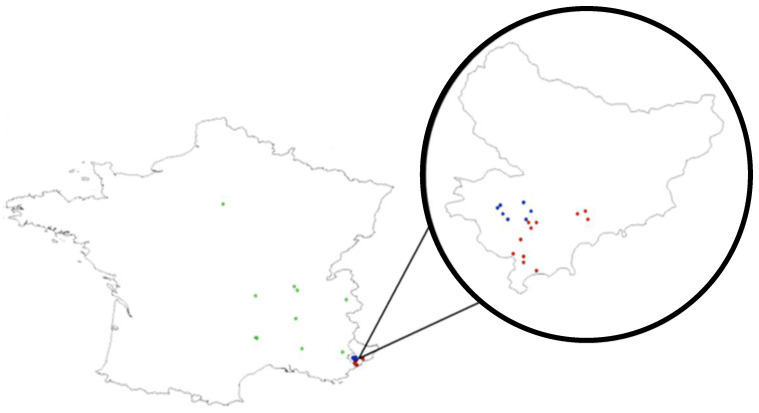
Map of the strains used this study. Left: overall view. Right: zoom of the French department, “Alpes-Maritimes”. The three strains’ origins are visualized as follows: meso-editerranean in red, supra-Mediterranean in blue and northwestern in green.

For this experiment, 40 strains of *T. cacoeciae* originating from south-eastern France were used (Table 1 and Figure 1). Here, the term “strain” refers to a group of wild-caught females (hereafter referred as to the “founders”) collected in the same location, in the same micro-habitats (same plant and hosts’ patch), at the same time and from which a perennial laboratory offspring has been obtained. These strains were representative of three geographic and climatic contexts, hereafter referred as to the “origins”. Three origins were distinguished here: “southern meso-Mediterranean”, “southern supra-Mediterranean” and “northwestern” (see details below).

### 2.2. Field Collection and Laboratory Rearing

Thirty strains were initiated from founders (about ten/strain, all these individuals being stored after their reproduction for the molecular characterization, see Section 2.3 § below) collected between 2016 and 2017 in a narrow geographic area (latitudes between 43.56° N and 43.80° N, longitudes between 6.84° E and 7.19° E) in south-eastern France (Department of “Alpes-Maritimes”, hereafter “southern”). This area is characterized by a marked altitudinal cline from the sea level to an altitude of 1500 m in less than 30 km. Based on climatic and ecological features [36], this altitudinal cline encompasses two bioclimatic zones, the meso-Mediterranean and the supra-Mediterranean, respectively represented here by 14 southern-meso strains and 16 southern-supra strains (see Table 1). The diversity and abundance of *Trichogramma* within this area is detailed elsewhere [34] but it is noteworthy that:

- Although there was an intense sampling effort [37] locations, and more than 9000 patches of the sentinel eggs of *Ephestia kuehniella* Zeller (Lepidoptera: Pyradidae) exposed], *Trichogramma* individuals were difficult to collect. On average, only 2% of hosts’ patches were actually parasitized.
- Among the eight recovered *Trichogramma* species, *T. cacoeciae* was the most abundant one and *T. evanescens* was the only species present all along the altitudinal cline.

The last 10 strains (hereafter, “Northwestern strains”) were sampled within a more northwestern area (latitudes between 43.79° N and 48.46° N, longitudes between 2.49° E and 6.63° E) characterized by more continental or even mountainous climates. All the strains of this last set were collected using sentinel eggs of *E. kuehniella* between 2015 and 2016 except the TCMZ strain which is an old reference strain (see Table 1). Because Northwestern strains were collected for the needs of a different project, less ecological information is available than for the southern ones. Moreover, the founders were not kept for molecular characterization (see § below), which was performed on the lab-reared offspring.

After sampling, all strains were then reared in standardized conditions within the Biological Resource Center “Egg Parasitoids Collection” (doi.org/10.15454/AY4LMT) using the substitution host *E. kuehniella*. Abiotic conditions were kept constant with a mean temperature of 18 °C ± 1 °C, a relative humidity of 75% ± 10%, and a photoperiod (light: dark) of 16 h:8 h (see for instance [26]). When the phenotyping for the thermal indices was initiated, most of the strains (main exception: TCMZ) had undergone between 15–30 generations in laboratory depending on their sampling date (see Table 1).

### 2.3. Molecular Characterization

This molecular characterization aims at discarding the existence of cryptic species that are frequent in Hymenopteran species [38,39,40,41] as well as the hypothesis of a phylogeographic process [37,42,43]—for instance, divergences at the molecular and phenotypic levels in an allopatric context followed by, after the expansion of some lineages, a secondary contact—that would better explain the observed patterns in thermal biology than processes of local adaptations. To achieve these goals, the mitochondrial gene cytochrome c oxidase subunit 1 (COI) was used for similar work (see [35,38]). It is worth noting that, *T. cacoeciae* being thelytokous, there is no genetic mixing including mito–nuclear introgression.

The molecular characterization was performed on founders (for the southern meso- and southern supra-Mediterranean strains) or on lab-reared offspring (three individuals) for Northwestern strains. Genomic DNA was extracted from recently killed individuals’ tissues using the prepGEM^®^ Insect kit (ZYGEM, PIN0500). Individuals were placed individually in 15 µL of mix and incubated for 3 h at 75 °C and then for 5 min at 95 °C. DNA extracts were stored at −20 °C.

A fragment (about 700 bp) was amplified using the primers LCO1490 and HCO2198 [44] and 1 µL of DNA was used for the PCR reaction (performed in a total volume of 25 µL). PCR was performed with the Multiplex PCR Master Mix QIAGEN (Cat No./ID: 206145) with a final concentration of 3 mM MgCl_2_ and 0.125 μL of each of the two primer solutions (100 μM). The PCR conditions were as follows: 95 °C for 15 min; 40 cycles at 95 °C for 30 s, 50 °C for 90 s, 72 °C for 1 min; and a final elongation step at 60 °C for 30 min. The size of PCR products was analyzed using a QIAxcel DNA Fast Analysis Kit (QIAGEN S.A.S) on a Qiaxcel Advanced System (QIAGEN S.A.S). PCR products were unidirectionally sequenced (primer HCO2198 only) using the Sanger method by the companies Beckman Coulter or Genewiz (Essex, GB).

The sequences obtained were then aligned and compared with a set of previously obtained COI haplotypes see Supplementary Materials Table S1 and [45]. A neighbor-joining tree was inferred using the Kimura 2-parameters distance and 500 replicates for bootstraps. All the analyses were performed using the MEGA software.

### 2.4. Thermal Tolerance Indices Phenotypic Characterization

As *Trichogramma* pre-imaginal life is parasitic and occurs within the host, phenotyping was performed on the mobile adults (less than 1 mm).

A prototype was especially designed and built for this experiment. This device allowed us to control the temperature of the air within a small circular arena (3 × 1 cm) where a small group of *Trichogramma* had been enclosed. Briefly, the cooling and warming were provided by a Peltier module controlled by a software developed for this purpose. The temperature of the arena was monitored in real time while the behavior of the individuals was observed from the top through a transparent wall and video-recorded using a digital video camera (Nikon D800 digital camera equipped with a 60 mm 1:2.8 G ED AF-S Micro Nikkor lens), the light being provided from the bottom.

After preliminary tests, each phenotyping session was done as follows: First, a group of about 50 young adults (i.e., emerged within the last 24 h) were transferred without anesthesia in the arena. The stage of “young adult” was used as only adults are mobile and as age may alter some thermal indices (David et al., 1998). Second, the temperature within the arena was initially set at +17 °C, a temperature at which the wasps were kept for no more than five minutes. Then, a progressive decrease of the temperature was controlled at a rate of 0.3 °C/ min until, in general, −2 °C (“routine program” on Table 1) and, sometimes, −4 °C (“extended program” on Table 1) was applied. Once the minimum was reached, an increase of the temperature occurred at the same rate until +14 °C. Each *T. cacoeciae* strain was phenotyped once. All the video-sequences were then carefully checked and only the behavior of the individuals (about 15–20 individuals) located on the floor of the arena and well visible were taken into account to estimate:

- Critical thermal minimum (CTmin): temperature at which the last individual lost its ability to walk;
- Chill coma temperature (CCT): temperature at which the first individual toppled with no more movement;
- Activity recovery (AR): temperature at which the first individual recovered its ability to walk.

### 2.5. Climatic Data

Climatic data were obtained from the French agency “Meteo France” (https://donneespubliques.meteofrance.fr/, accessed on 1 January 2019) for stations distributed between the three geographic origins: (i) southern-meso: three stations, (ii) southern-supra: three stations, and (iii) northwestern: nine stations (Table 2). For each of these stations, daily mean (Tmean) and minimal (Tmini) temperatures observed during the three coldest months (December−February) for three successive years (2015–2017) were extracted and averaged.

Hence:

- Tmean varied between +6.3 °C and +9.3 °C in the southern-meso, +2.2 °C and +4.8 °C in the southern-supra, and +1 °C and +6.6 °C in the northwestern.
- Tmini varied between +2.8 °C and +6.7 °C in the southern-meso, −0.5 °C and +1.5 °C in the Southern-Supra, and −4.7 °C and +2.1 °C in the northwestern.

### 2.6. Statistical Analyses

Statistical analyses were performed with the software R (version R-3.6.0) and its working environment Rstudio (Version 1.1.453).

The intraspecific differentiation was tested on each of the three thermal tolerance indices (CTmin, CCT, and AR) using two complementary approaches—an hypothesis test approach and a model comparison approach realized with linear mixed effects models (LME models). Two sets of explanatory variables were investigated separately:

- The first set of variables (hereafter, the hypothesis test approach) included the “origin” (qualitative variable with three modalities: southern-meso, southern-supra, and northwestern) as the only fixed effect. The sampling location and the COI haplotype were concatenated into a single qualitative variable and used as a random effect. This was to prevent any pseudo-replication linked to the use of strains deriving from the same “natural population” (see Field Collection and Laboratory Rearing).
- The second set of variables (hereafter, the model comparison approach) considered the two climatic variables (Tmean and Tmini—see previous section) as well as three geographic ones (altitude, latitude, and longitude) as fixed effects. These quantitative variables thus substituted the qualitative variable “origin” used in the first analysis. As for the first analysis, the concatenated information about the sampling location and the COI haplotype was used as a first random effect (intercept). Insofar as several strains are linked to the same meteorological station, this information was used as a second random effect (intercept).

In both cases (the hypothesis test approach and the model comparison approach), the analysis started with the selection of random effects based on in the first case on the Akaike information criteria (AIC) and in the second case on the conditional Akaike information criteria (cAIC) [46,47]. 

For each trait, all the models, i.e., the null one, the model with “origin” as the sole fixed factor in hypothesis test approach, and the 21 models obtained with the model comparison approach—were compared (models leading to singularity being discarded) and those minimizing the information criterion were conserved. If fixed effects were involved, they were tested using likelihood ratio tests (LRT) or Fisher’s exact test (see Table 3). 

For each thermal index (respectively, CTmin, CCT, and AR) and each approach (hypothesis test and, when relevant, model comparisons—see Materials and Methods), the model selection was realized using the Information Criteria (see Supplementary Materials Table S2). The selected fixed factors were tested using likelihood ratio test (LRT) and/or Fisher’s exact test (*f*-value).

For significant qualitative variables, post-hoc Tukey tests were also performed. Several R packages were used (in particular cAIC4, multcomp, effects, and ggplot) for the statistical analysis or graphic representations.

In order to explore possible correlations between thermal tolerance indices, pairwise Spearman’s nonparametric rank correlation tests were performed to test possible correlations between the three thermal tolerance indices. Then, a principal component analysis (PCA) was carried out after the normalization of the data in order to graphically visualize the between-trait correlations as well as individual and mean performances (R packages: FactoMineR and Factoextra). 

## 3. Results

### 3.1. Molecular Characterization Using COI

**Figure 2:**
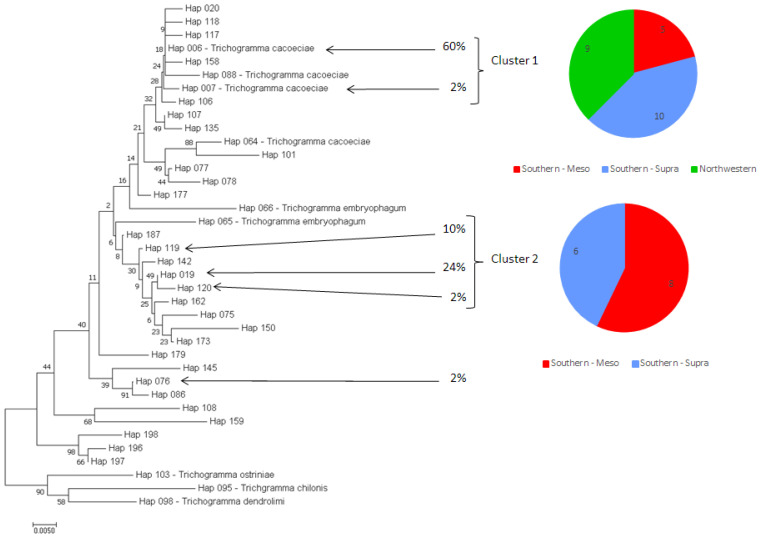
Neighbor-joining tree obtained using part of the mitochondrial gene COI. The evolutionary distances were computed using the Kimura 2-parameter method using 500 replicates for bootstrap tests. The final set included a total of 272 positions, all ambiguous positions having been previously removed for each sequence pair. Percentages indicate the frequencies of the haplotypes within the strains used in this study.

The 40 strains were characterized using part of the COI. As shown in Figure 2, six valid haplotypes were obtained from the 40 strains, three (“Hap 006”, “Hap 019” and “Hap 119”) of them representing 94% of the strains and the other ones being observed only once. 

Based on the neighbor-joining tree, two clusters could be distinguished, Cluster 1 (“Hap 006” and “Hap 007”) and Cluster 2 (“Hap 019”, “Hap 119” and “Hap 120”). The frequencies of these two clusters did not seem be independent from the “origins” of the strains since the relative proportions of the Cluster 1 were 90%, 73%, and only 33% within respectively “northwestern”, “southern-supra”, and “southern-meso” origins (Fisher’s exact test: *p*-value = 0.011).

### 3.2. Behavior of the Wasps at Cold Temperatures

**Figure 3:**
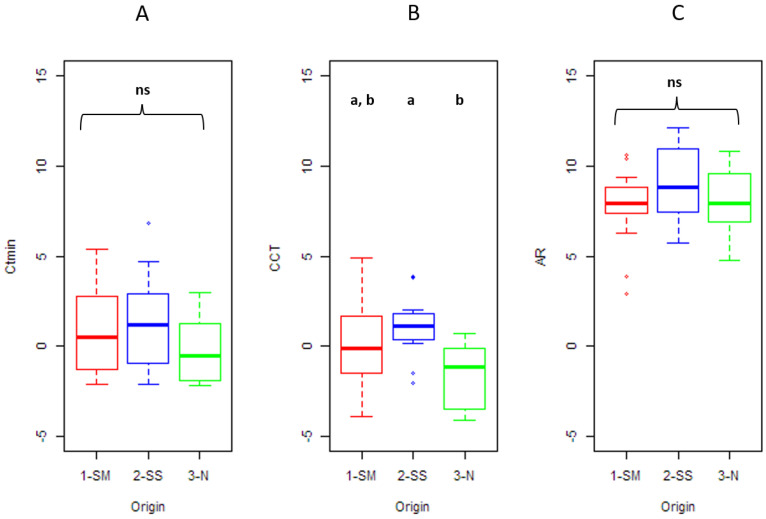
Influence of the variable “Origin” on the three thermal tolerance indices: CTmin (**A**), CCT (**B**) and AR (**C**).

When the temperature decreased, all strains lost, at some point, their ability to move their limbs in a coordinated manner and were therefore unable to walk. At colder temperatures, individuals started to topple on one of their sides with no more movement. Within a pool of individuals, there was, however, a large overlap between individual CTmin and CCT what explains the following results. Indeed, the median value for the last individual reaching CTmin was 0 °C with an interquartile between −1 °C and 0 °C. The median value for the first individual reaching CCT was +1°C with an interquartile between 0 °C and +1 °C. Then, when temperature increased, the mobility was restored. The median value for the AR was +9 °C with an interquartile of +8.50 °C and +9 °C. AR values have to be treated with caution insofar as, the CCT varying between strains, the strains had variable exposures to cold temperatures before the warming. No mortality was observed during the phenotyping. Figure 3 more precisely represents the distributions of each of the three thermal tolerance indices according to the three geographical origins (northwestern, southern meso-Mediterranean, and outhern supra-Mediterranean).

### 3.3. Genetic Differentiation between Geographic Origins 

The hypothesis test approach (“origin” as the sole explanatory variable) did not allow detecting relevant factors for CTmin and activity recovery (Table 3 and Figure 3). By contrast, evidence for spatial differentiation was observed for the chill coma temperature, the fixed effect “origin” being significant. More precisely, a significant difference was observed between the “Northwestern” origin (median of −1.4 °C) and the “southern supra-Mediterranean” one (median of −0.4°C) (z = 3.256; *p* = 0.003). No significant difference was observed for the two other comparisons, “northwestern” versus “southern meso-Mediterranean” (z = 2.090, *p* = 0.073) or “southern meso-Mediterranean” versus “southern supra-Mediterranean” (z = 1.260, *p* = 0.208).

For each thermal tolerance index, the boxplot indicates the median, the interquartile, the minimums and maximums as well as any values considered extreme. For each index, the significance of the variable “origin” is provided in Table 3 (Hypothesis test approach). Results of post hoc tests are represented by different letters (a and b to indicate a significant difference and ns to indicate a non-significant difference). Boxplot in red, blue and green color respectively represent the three geographic origins: southern meso-Mediterranean strains (SM), southern supra-Mediterranean (SS), northwestern strains (N). 

**Figure 4:**
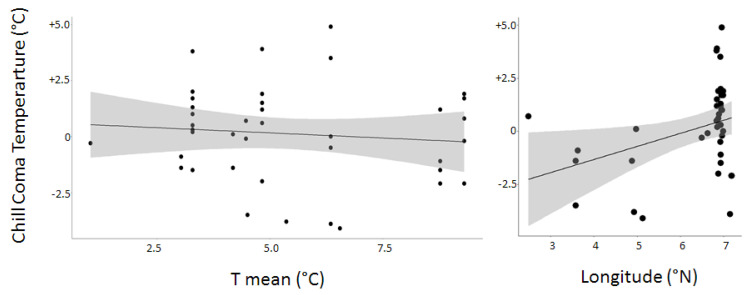
Influence of the climatic variable “Tmean” and the geographic variable “Longitude” on the chill coma temperature. The points, linear trend, and gray ribbon, respectively, represent the raw data and the predicted regression as well as the 95% confidence interval. The statistical significance of these two variables is indicated in the Table 3.

The model comparison approach gave different outcomes at the step of model selection according to the thermal index considered (see Supplementary Materials Table S2). For CTmin (linear mixed effects model), the values of cAIC were very close to each other (from 104.08 to 104.30) and all the models are equivalent to each other in information and to the null model. For the activity recovery, the values of AIC were close (from 172.3 to 181.2) and the best model was the null one, i.e., those without fixed effects. For the chill coma temperature (linear mixed effects model), the values of CAIC were far more variable (from 179.13 to 226.02), the best model being those involving three fixed effects: the daily mean temperature observed during the three coldest months (Tmean), the daily minimal temperature observed during the same period (Tmini), and the longitude (cf. Table 3). Two of these (Tmean and longitude) were proven significant, the chill coma temperature being negatively correlated with the mean temperature during the coldest months (Tmean) and positively with the longitude (Table 3 and Figure 4). This thus confirms the results of the hypothesis test approach.

### 3.4. Pairwise and Multivariate Correlations between Thermal Tolerance Indices 

Among the three pairwise correlations between CTmin, CCT, and AR, only those between CTmin and CCT were significant and highly positive (Spearman’s rank correlation tests: rho = 0.43, *p*-value = 0.006). On the contrary, no correlation was found between AR and CCT (rho = −0.14, *p*-value = 0.38) or between AR and CTmin (rho = −0.11, *p*-value = 0.52). 

**Figure 5:**
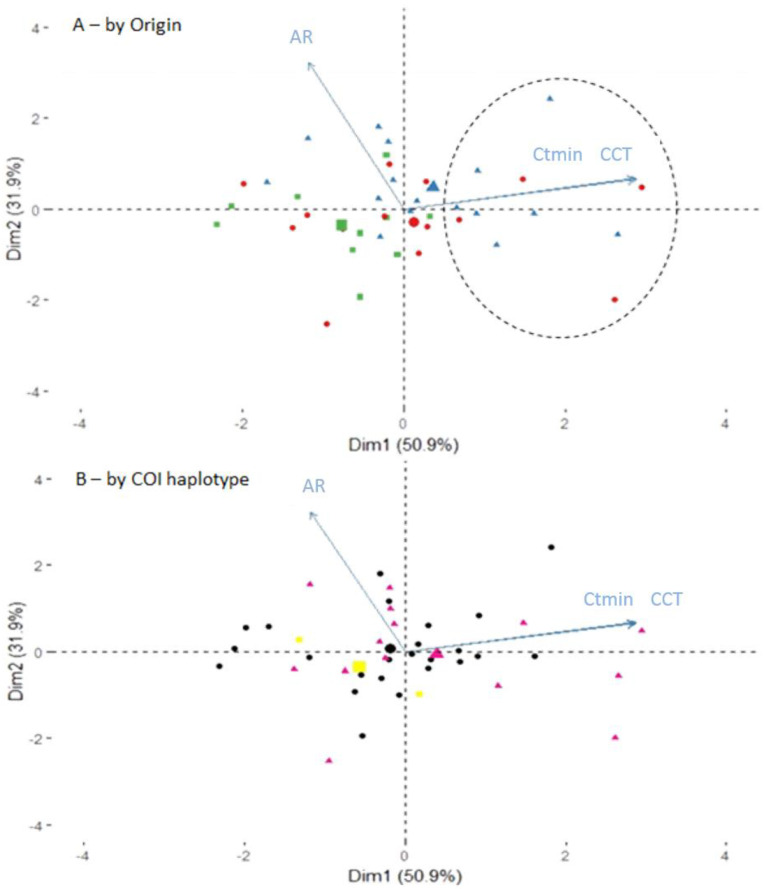
Principal component analysis (PCA) performed on the three thermal tolerance indices (CTmin, CCT, and AR). The PCA was performed after the normalization of the three variables. The projected inertia is provided for each axis. (**A**) Visualization according the origin of the strains. Red circles, blue triangles, and green squares, respectively, represent southern meso-Mediterranean, southern supra-Mediterranean, and northwestern strains, bigger symbols indicate the mean position for each origin. The dotted ellipse includes southern strains with highest values on the first axis of the PCA. (**B**) Visualization according the molecular characterization. Black circles, pink triangles, and yellow squares, respectively, represent cluster 1, cluster 2, and other haplotypes (see Figure 1), bigger symbols indicate the mean position for each haplotype.

To further investigate possible covariations between these three traits, a principal component analysis (PCA) was performed on normalized data, the first two axes explaining 83% of the variability (Figure 5). Most of the variability on the first axis was explained by the positive correlation between CTmin and CCT while most of the variability on the second axis was explained by the AR. Within this phenotypic space, both the meso- and supra-Mediterranean strains appeared to be more variable than the Northwestern ones. In particular, 29% of the meso-Mediterranean strains and 44% of supra-Mediterranean ones exhibited high values on the first PCA axis (see dotted ellipse on Figure 5A). Such high values were not encountered in the northwestern strains. As shown in Figure 5B, differences in phenotypic performances did not depend on the COI haplotypes.

## 4. Discussion

Our study evidences two main findings, the existence of a significant geographic differentiation for the chill coma (CC) and the positive correlation between the chill coma (CC) and critical thermal minimum (CTmin). Because the strains were reared during several generations in standardized conditions before their phenotyping, these two patterns are unlikely to be explained by (trans-generational) plastic responses. They thus rather reflect an actual intra-specific genetic variability. It is also noteworthy that the two patterns cannot be linked with the molecular clustering observed on COI. Indeed, based on surveys in other areas [45], the COI haplotype “Hap 006” (the most frequent one in cluster 1) incidentally appears widely distributed in Europe and also present in North Africa which suggests an ancient expansion from its original area. With such a wide geographic distribution, it is thus not surprising that contrasting life-history strategies may have been selected according to contrasting local conditions. So far, the COI haplotype “Hap 019” appears to be less cosmopolitan and, to date, only observed in the south-east of France. This does not however prevent this haplotype from including a wide range of phenotypes. Although the use remains, of course, of limited relevance to investigate population genomics, we can, however, discard the hypothesis that the phenotypic differentiation between bioclimatic zones is explained by different ancient lineages. We would thus rather explain the two patterns (significant differentiation on CC and positive correlation between CC and CTmin) as the consequence of local adaptions. As highlighted in Supplementary Materials Table S2, this study thus actually contributes to filling the deficit of knowledge about the intraspecific variability of thermal tolerance indices in insects and, in particular, in parasitoids.

Under temperate, mountainous, or even polar climates, the survival of ectotherms to low temperatures is a central question in understanding their ecology and evolution [48]. According to (i) the intensity and duration of the cold, (ii) the species’ biology, and (iii) the stage of the development, various behavioral and/or physiological responses are observed including the research of local favorable micro-habitats, migration towards more favorable locations, “dormancy” (diapause or quiescence), and/or an improved tolerance [49,50]. With respect to this last point, several authors emphasize the relevance of the three thermal tolerance indices, critical thermal minimum [9], chill coma temperature, and activity recovery [15,16,17,18,19,20,21,22,23,24,25,26,27,28,29,30,31,32,33,34,35,36,37,38,39,40,41,42,43,44,45,46,47,48,49,50,51] as relevant proxies of the adaptation to low temperatures. As proxies, the mean values per geographic origins (meso-Mediterranean, supra-Mediterranean, and northwestern) observed for each thermal tolerance index are not ecologically meaningful per se but the ranking between the three origins (e.g., for CC: northwestern < meso-Mediterranean < supra-Mediterranean) is informative. This same reasoning was already held by Andersen and colleagues [9] and Le Laan and colleagues [52] at an interspecific level. 

At the intraspecific level, several studies evidenced some strain differentiation according to latitude [53] and/or altitude. Nevertheless, the pervasiveness of such patterns and the scale at which they occur remain unclear. Our understanding of such processes is also limited by the lack of information about the potential genetic correlations between these thermal tolerance indices as well as between these indices and other facets of the adaptation to cold temperatures (e.g., lowest temperature for the development or propensity to diapause) although this aspect is mandatory to understanding possible constraints or synergies between traits [53]. In fact, the few references we have found about correlations between thermal tolerance indices deal with interspecific comparisons [9,10] or with environment-induced phenotypic variations [54]. The extrapolation of such results at the intra-specific level remains unreliable.

Within this frame, we thus addressed the question of the spatial scale at which strain differentiation and local adaptations may occur by considering both a macro- (“southern” versus “northwestern” strains) and a micro- (within southern strain, “meso-” versus “supra-Mediterranean” strains) scales. Taken as whole, our results evidenced a phenotypic differentiation at the macro-scale with a significant effect of both the longitude and the severity the cold during winter. This led to a significantly lower median for the CCT in northwestern strains than in the southern supra-Mediterranean ones. Because environmental conditions were standardized during the phenotyping and, before, during several generations of lab-rearing, this differentiation can only be explained by genetic differences. This result was quite counter-intuitive insofar as we expected similar trends for these two origins for which similar cold climates are observed during winter. The reasons why the micro-process (genetic differentiation along the altitude cline) differ from the macro-process (genetic differentiation along the latitudinal/longitudinal one) are not understood. Discrepancies between the effect of, on one side, altitude, and, on the other, latitude, were however already observed for other traits and species [55,56,57,58]. Moreover, the strong correlation between the CCT and the CTmin suggests either independent but convergent selections on these two traits, or a genetic (pleiotropy) or physiological linkage [59,60].

A future direction of this study is to complete the phenotyping of these strains using other traits involved in the adaptation to cold temperatures. Based on the literature on *Trichogramma* species, two complementary facets are thus planned to be investigated—overwintering strategies in field conditions [61,62,63], and propensity to diapause in laboratory conditions [64,65]. The objective will, hence, be to investigate if global strategies for low temperature exist and, if so, what could be the possible trade-off with other parts of the thermal range, the “comfort zone” or sub-lethal warm temperatures [46,66,67]. In addition to their relevance for fundamental research, such results might open major perspectives for the genetic improvement of *Trichogramma* strains used as biological control agents.

## Figures and Tables

**Table 1:** Summary of reference *Trichogramma cacoeciae* strains used for the phenotyping characterization and temperature values of CTmin, CCT, and AR.

Site	Reference Strain	Haplotypes	Cluster	Origin	Lat	Long	Alt	Month	Year	Programme	Ctmin	CCT	AR	Code Weather Stations	Code Meteo France Stations
S01	ISA14032	Hap119	Cluster 2	southern-meso	43.77	7.18	178	November	2016	1	−2.1	−2.1	3.9	H	6152002
S02	ISA17128	Hap006	Cluster 1	southern-meso	43.76	7.15	667	June	2016	1 & 2	−0.5	−3.9	10.6	G	6118002
S05	ISA19503	Hap019	Cluster 2	southern-meso	43.74	7.19	41	September	2016	1	−1.5	−2.1	NA	I	6029001
S13	ISA17127	Hap019	Cluster 2	southern-meso	43.56	6.99	142	October	2016	1	4.2	1.9	8.8	H	6152002
S13	ISA15034	Hap020	Other	southern-meso	43.56	6.99	142	November	2016	1	−1.3	1.7	6.3	H	6152002
S16	ISA17141	Hap006	Cluster 1	southern-meso	43.59	6.94	13	October	2016	1	1.6	1.2	7.5	I	6029001
S17	ISA7271	Hap006	Cluster 1	southern-meso	43.61	6.94	59	June	2016	1	−1.3	−1.5	8.8	I	6029001
S19	ISA15220	Hap006	Cluster 1	southern-meso	43.61	6.94	68	November	2016	1	2.8	−1.1	7.4	I	6029001
S20	ISA4561	Hap120	Cluster 2	southern-meso	43.62	6.90	201	April	2016	1	0.4	0.8	10.4	H	6152002
S21	ISA17138	Hap019	Cluster 2	southern-meso	43.67	6.93	564	october	2016	1	0.6	−0.5	8.2	G	6118002
S21	ISA13580	Hap119	Cluster 2	southern-meso	43.67	6.93	564	October	2016	1	3.6	3.5	2.9	G	6118002
S22	ISA6064	Hap006	Cluster 1	southern-meso	43.71	6.97	261	May	2016	1	2.7	−0.2	9.4	H	6152002
S23	ISA17658	Hap019	Cluster 2	southern-meso	43.73	6.99	684	April	2017	1	−1.9	0	7.9	G	6118002
S24	ISA17135	Hap019	Cluster 2	southern-meso	43.73	6.96	848	April	2016	1	5.4	4.9	7.6	G	6118002
S26	ISA17130	Hap006	Cluster 1	southern- supra	43.74	6.95	1089	April	2016	1	−1.7	1	7.3	F	6050002
S26	ISA17129	Hap119	Cluster 2	southern- supra	43.74	6.95	1089	April	2016	1	−0.2	1.7	11.4	F	6050002
S29	ISA10488	Hap006	Cluster 1	southern- supra	43.74	6.88	1139	August	2016	1 & 2	−1.7	−2	10.5	D	6037002
S29	ISA17139	Hap119	Cluster 2	southern- supra	43.74	6.88	1139	August	2016	1	−2	1.9	9	D	6037002
S29	ISA17140	Hap119	Cluster 2	southern- supra	43.74	6.88	1139	August	2016	1	−2.1	0.6	12.1	D	6037002
S31	ISA11754	Hap006	Cluster 1	southern- supra	43.76	6.86	1094	September	2016	1	2.9	0.2	8	F	6050002
S32	ISA19505	Hap019	Cluster 2	southern- supra	43.77	6.97	700	October	2016	1	0	1	9.7	F	6050002
S34	ISA11785	Hap006	Cluster 1	southern- supra	43.78	6.84	985	September	2016	1	1.1	0.5	8.6	F	6050002
S34	ISA19192	Hap006	Cluster 1	southern- supra	43.78	6.84	985	May	2016	1 & 2	0.4	3.8	9.5	F	6050002
S35	ISA14786	Hap006	Cluster 1	southern- supra	43.79	6.85	1187	November	2016	1	4.7	3.9	12.1	D	6037002
S35	ISA18282	Hap006	Cluster 1	southern- supra	43.79	6.85	1187	October	2016	1	4.4	1.5	7.2	D	6037002
S35	ISA13744	Hap019	Cluster 2	southern- supra	43.79	6.85	1187	October	2016	1	2.6	1.2	6.1	D	6037002
S37	ISA17124	Hap006	Cluster 1	southern- supra	43.80	6.94	878	June	2016	1	2.3	1.3	7.6	F	6050002
S37	ISA17125	Hap006	Cluster 1	southern- supra	43.80	6.94	878	June	2016	1	1.3	0.3	12.1	F	6050002
S37	ISA17954	Hap006	Cluster 1	southern- supra	43.80	6.94	878	April	2017	1	2.9	−1.5	8.2	F	6050002
S37	ISA17940	Hap019	Cluster 2	southern- supra	43.80	6.94	878	April	2017	1	6.8	2	5.7	F	6050002
S51	ACJYR0144	Hap006	Cluster 1	northwestern	45.55	6.63	735	July	2015	2	−0.1	−0.1	6.4	A	91184001
S52	ACJYR0031	Hap006	Cluster 1	northwestern	48.46	2.49	75	June	2015	1 & 2	−2.1	0.7	7.6	A	91184001
S53	FLO0064	Hap006	Cluster 1	northwestern	44.39	3.58	1225	June	2016	2	−1	−1.4	4.8	B	48186001
S54	FLO239	Hap006	Cluster 1	northwestern	44.38	3.63	1074	June	2016	2	−0.9	−0.9	6.9	B	48186001
S55	GOT113A	Hap076	Other	northwestern	44.97	4.93	171	May	2016	2	1.3	−3.8	9.6	C	26064001
S56	ISA1067	Hap006	Cluster 1	northwestern	45.95	4.88	290	Unknown	2015	2	3	−1.4	10.8	E	1027003
S57	ISA1075	Hap006	Cluster 1	northwestern	45.83	4.98	299	Unknown	2015	2	0	0.1	8.1	E	1027003
S58	SALA001	Hap006	Cluster 1	northwestern	43.96	6.50	917	July	2017	2	2.2	−0.3	7.8	L	4136001
S59	TCMZ	Hap007	Cluster 1	northwestern	44.06	5.13	151	Unknown	1987	2	−2.2	−4.1	9	M	84031001
S60	TSM008	Hap006	Cluster 1	northwestern	45.67	3.58	592	July	2015	2	−1.9	−3.5	9.7	N	63125002

**Table 2:** Summary of reference weather stations and daily mean (average T) and minimal (minimum T) temperatures observed during the three coldest months (December−February).

Stations	Code Meteo France Stations	Origin	Lat	Long	Altitude	Average T	Minimum T
Cannes	6029001	southern—meso	43.556	6.95	2	8.77	4.58
Valbonne	6152002	southern—meso	43.623	7.028	238	9.3	6.65
St. Cezaire sur Siagne	6118002	southern—meso	43.678	6.809	694	6.35	2.81
Coursegoules	6050002	southern-supra	43.792	7.048	985	3.29	0.38
Caussol	6037002	southern-supra	43.752	6.923	1268	4.83	1.48
Les Mas	6081001	southern-supra	43.813	6.809	1525	2.21	−0.54
Aiguines	83002004	northwestern	43.775	6.245	853	5.39	1.24
La Mure Argens	4136001	northwestern	43.775	6.245	920	1.04	−4.66
Carpentras	84031001	northwestern	43.977	6.52	109	6.56	2.07
La Salle Prunet	48186001	northwestern	44.075	5.059	1030	3.04	0.2
Valence—Chabeui	26064001	northwestern	44.914	4.971	163	5.37	1.9
Bourg St. Maurice	73054001	northwestern	44.315	3.65	864	1.19	−2.12
Balan	1027003	northwestern	45.754	3.572	194	4.19	0.73
Courpiere	63125002	northwestern	45.612	6.763	460	4.51	1.48
Courdimance	91184001	northwestern	45.833	5.105	67	4.48	1.31

**Table 3:** Statistical significance of fixed factors.

A—CTmin	Variable	SS	MS	DF1	DF2	*f*-Value	*p*-Value
Hypothesis test	Origin	0.64	0.32	2	32.0	1.01	0.377
**B—Chill Coma Temperature**	**Variable**	**SS**	**MS**	**DF1**	**DF2**	***f*-Value**	***p*-Value**
Hypothesis Test	Origin	33.51	16.75	2	27.95	5.32	0.011 *
	**Variable**				**AIC**	**LRT**	***p*-Value**
Model Comparison	T_mean T_mini Longitude				176.41 175.24 179.67	4.06 2.89 7.31	0.044 * 0.089 0.009 **
**C—Activity Recovery** Hypothesis Test	**Variable** Origin	**SS** 15.07	**MS** 7.54	**DF1** 2	**DF2** 36	***f*-Value** 1.74	***p*-Value** 0.190

For each thermal index (respectively CTmin, CCT and AR) and each approach (Hypothesis test and, when relevant, Model comparisons—see Material and Methods), the model selection was realized using the Information Criteria (see Supplementary Materials Table S2). The selected fixed factors were tested using Likelihood Ratio Test (LRT) and/or Fisher tests (*f*-value). The significant *p*-values are marked with the asterisks.

## Data Availability

Data are available in article’s Supplementary Materials.

## Supplementary Materials

The following are available online at https://www.mdpi.com/article/10.3390/insects12111013/s1 (the link will be generated): Table S1: Summary of reference strains used for the molecular characterization of the *Trichogramma cacoeciae* used in this study. Haplotypes corresponding to the strains used in this study are indicated in bold. The columns “reproduction” and “amino-acid sequence” indicate, respectively, the mode of reproduction of the strain (A = arrhenotoky, T = thelytoky) and the variation in the corresponding amino-acid sequence (label “varprot”) and thus putative pseudogene or technical artefacts. Upper part: strains available at the Biological Resource Center “Egg Parasitoids Collection” (doi.org/10.15454/AY4LMT) (source: Warot 2018). Identification was formerly realized by B. Pintureau. Lower part: Genbank references. Table S2: Summary of the statistical models evaluated in the study. According to the selection of the random effects, the nature of the model was either a linear mixed effects model (LME) or a linear model (LM). For each of the traits, the two approaches are presented, i.e., the test hypothesis (“Origin” as the sole fixed effect) and the model comparison. In this second case, the null model and the 31 possible combinations obtained from the two meteorological variables (t_mean and t_mini) and the geographic ones (alt, lat, and lon) are provided. The model minimizing the Information criteria (cAIC for LME and AIC for LM) is indicated in bold.
