# Supplementary material for: Genetic Variability, Population Differentiation, and Correlations for Thermal Tolerance Indices in the Minute Wasp, Trichogramma cacoeciae
Source: Insects. 2021 Nov 10;12(11):1013. doi: 10.3390/insects12111013 (PMC8622974; doi:10.3390/insects12111013)
Supplement: Supplementary file 1 [file insects-12-01013-s001.zip › insects-1372678-supplementary.pdf]

Supplementary materials

Supplementary Materials Table S1: Summary of reference strains used for the molecular characterization of the *Trichogramma cacoeciae* used in this study.

Haplotypes corresponding to the strains used in this study are indicated in bold.

The columns "reproduction" "amino-acid sequence" respectively indicate the mode of reproduction of the strain (A=Arrhenotoky – T=Thelytoky) as well as variation in the corresponding amino-acid sequence (label "varprot") and thus putative pseudogene or technical artefacts.

Upper part: strains available at the Biological Resource Center "Egg Parasitoids Collection" (doi.org/10.15454/AY4LMT)(source : Warot 2018). Identification were formely realized by B. Pintureau.

Lower part : Genbank references

Haplotypes	Reference strain	Strain's origin	Identification	Reproduction	GENBANK Accession	Amino-acid sequence
Information obtained from the BRC EP-Coll						
**Hap_006**	**TCAL**	**France, 1987-1989**	***T. cacoeciae***	**T**	**MG932142**	
**Hap_007**	**TCMz**	**France, 1987-1989**	***T. cacoeciae***	**T**	**MG932143**	
**Hap_019**	**PMBio1**	**France, 2014**	**_**	**T**	**MG932155**	
**Hap_020**	**ISA15034**	**France, 2016**	**_**	**T**	**MG932156**	**varprot**
Hap_064	101	France, 1976	*T. cacoeciae*	T	MG932198	
Hap_065	URO2	Iran, 1996	*T. embryophagum*	A	MG932199	
Hap_066	URO3	Iran, 1996	*T. embryophagum*	A	MG932200	
Hap_075	Grab_haplo7	France, 2011	_		MG932209	
Hap_076	GOT0098	France, 2016	_		MG932210	
Hap_077	ISA11030	France, 2016	_		MG932211	
Hap_078	Grab_haplo10	France, 2011	_		MG932212	
Hap_086	COR10 sp1	France (Corsica), 2016	_		MG932216	
Hap_087	_	_	_		KM242285	varprot
Hap_088	_	_	_		DQ177917	
Hap_101	ACJYR0132	France, 2015	_		MG932224	varprot

Hap_102	_	_	_		KM105169	varprot
Hap_106	ACJYR0116	France, 2015	_		MG932227	
Hap_107	ACJYR0121	France, 2015	_		MG932228	
Hap_108	NS008	France, 2015	_	A	MG932229	
Hap_117	ESP227	Spain, 2016	_		MG932236	
Hap_118	PMBIO2	France, 2014	_		MG932237	
**Hap_119**	**ISA4737**	**France, 2016**	**_**	**T**	**MG932238**	
**Hap_120**	**ISA4561**	**France, 2016**	**_**	**T**	**MG932239**	
Hap_135	COR7	France (Corsica), 2016	_		MG932253	
Hap_142	ISA6151_R2.6	France, 2016	_		MG932260	
Hap_145	GOT0098	France, 2016	_		MG932262	
Hap_150	ISA12949	France, 2016	_		MG932267	
Hap_158	ISA11783	France, 2016	_		MG932274	
Hap_159	ISA12953	France, 2016	_		MG932275	
Hap_162	ISA15036	France, 2016	_		MG932277	
Hap_173	FLO218	France, 2016	_		MG932282	
Hap_177	ISA7851_9	France, 2016	_		MG932285	
Hap_179	ISA11365_2	France, 2016	_		MG932287	
Hap_187	ISA10468.2	France, 2016	_		MG932295	
Hap_196	Pch1	Algeria, 2017	_	A	MG932303	
Hap_197	Pch1	Algeria, 2017	_	A	MG932304	
Hap_198	Pch2	Algeria, 2017	_	A	MG932305	
Complementary data obtained from Genbank						
Hap_088	_	_	*T. cacoeciae*		DQ177917	
Hap_095	_	_	*T. chilonis*		DQ177915	
Hap_098	_	_	*T. dendrolimi*		KC411494	
Hap_103	_	_	*T. ostriniae*		DQ177914	

Supplementary Materials Table S2: Summary of the statistical models evaluated in the study.

According to the selection on the random effects, the nature of the model was either a Linear Mixed Effects model (LME) or a Linear model (LM). For each of the traits, the two approaches were presented, *i.e.* the Test hypothesis ("Origin" as the sole fixed effect) and the Model comparison. In this second case, the null model and the 31 possible combinations obtained from the two meteorological variables (t_mean and t_mini) and the geographic ones (alt, lat and lon) are provided. The model minimizing the Information criteria (cAIC for LME and AIC for LM) is indicated in bold.

**Trait**	**Approach**	**Parameters**	**Origin**	**t_mean**	**t_mini**	**alt**	**lat**	**lon**	**cAIC /AIC**
**CTmin** (LME - site x haplotype as random factor)									
	Test hypothesis	1 parameter	X						104.19
	Model comparison	Null model							104.11
		1 parameter		X					104.20
					X				104.20
						X			104.19
							X		104.14
								X	104.08
		2 parameters		X	X				104.27
				X		X			104.25
				X			X		104.21
				X				X	104.12
					X	X			104.26
					X		X		104.22
					X			X	104.13
						X	X		104.21
						X		X	104.14
							X	X	104.18
		3 parameters		X	X	X			104.30
				X	X		X		104.29
				X	X			X	104.20
				X		X	X		104.23
				X		X		X	104.16

				X			X	X	104.21
					X	X	X		104.27
					X	X		X	104.20
					X		X	X	104.23
						X	X	X	104.23
		4 parameters		X	X	X	X		104.28
				X	X	X		X	104.21
				X	X		X	X	104.29
				X		X	X	X	104.24
					X	X	X	X	104.28
		5 parameters		X	X	X	X	X	104.28
**Chill Coma Temperature** (LME - site x haplotype as random factor)									
	Test hypothesis	1 parameter	X						183.96
	Model comparison	null model							180.77
		1 parameter		X					182.77
					X				182.05
						X			225.90
							X		183.47
								X	183.07
		2 parameters		X	X				182.79
				X		X			_
				X			X		186.33
				X				X	183.63
					X	X			_
					X		X		185.61
					X			X	185.32
						X	X		226.02
						X		X	198.19
							X	X	183.43

		3 parameters		X	X	X			_
				X	X		X		184.58
				**X**	**X**			**X**	**179.13**
				X		X	X		_
				X		X		X	204.65
				X			X	X	184.17
					X	X	X		_
					X	X		X	_
					X		X	X	185.65
						X	X	X	_
		4 parameters		X	X	X	X		207.95
				X	X	X		X	186.15
				X	X		X	X	179.89
				X		X	X	X	_
					X	X	X	X	_
		5 parameters		X	X	X	X	X	194.50
**Activity Recovery** (LM – no random factor)									
	Test hypothesis	1 parameter	X						172.75
	Model comparison	null model							**172.35**
		1 parameter		X					173.86
					X				173.88
						X			174.00
							X		174.34
								X	174.15
		2 parameters		X	X				175.86
				X		X			175.84
				X			X		175.82
				X				X	175.43
					X	X			175.84

		X		X		175.84
		X			X	175.46
			X	X		175.98
			X		X	175.87
				X	X	175.89
3 parameters	X	X	X			177.84
	X	X		X		177.81
	X	X			X	177.43
	X		X	X		177.82
	X		X		X	177.37
	X			X	X	177.19
		X	X	X		177.83
		X	X		X	177.46
		X		X	X	177.22
			X	X	X	177.42
4 parameters	X	X	X	X		179.81
	X	X	X		X	179.37
	X	X		X	X	179.19
	X		X	X	X	179.19
		X	X	X	X	179.21
5 parameters	X	X	X	X	X	181.19
